# Combining three‐dimensional histopathology with bread loafing and orientation without artificial coloring

**DOI:** 10.1111/cup.14239

**Published:** 2022-04-27

**Authors:** Nina Zila, Philipp Tschandl

**Affiliations:** ^1^ Department of Dermatology Medical University of Vienna Vienna Austria

## 1 INTRODUCTION

Basal cell carcinoma (BCC), one of the subtypes of non‐melanoma skin cancer (NMSC), is the most common cancer of the skin, and its incidence is increasing worldwide.^1^ BCCs rarely metastasize but can cause significant morbidity because of local invasion and tissue destruction. In up to 80% of patients, BCCs develop in the head and neck region, and surgical removal is generally considered the treatment of choice.^2^ Given the proximity of many sensitive anatomic landmarks, there is a need for resection margin control. Especially in small and narrow excisions, full margin work‐up can potentially deteriorate the quality of the center of the specimen, leaving little to no tumor to evaluate architecture and pattern. While Mohs micrographic surgery is recommended for critical areas, such as the central part of the face, and has been shown to ensure low recurrence rates,^3^, ^4^ this technique is often cumbersome and complicated for non‐specialized clinics. Mohs procedures need to be performed by surgeons with special training, and the procedure itself can take several hours because of removing layer after layer until skin samples are free of cancer cells. On the contrary, excising the tumor with primary or delayed closure, and subsequent macroscopic bread loafing the specimen by a pathologist is a much easier procedure. This technique allows better evaluation of both architecture and pattern, but only limited assessment of the margin. The sensitivity in detecting residual tumor at the surgical margins of facial BCCs, excised with 2‐mm surgical margins and bread loafing at 4‐mm intervals, was reported to be only 44%.^5^ Therefore, to reduce the risk of tumor recurrence and overcoming the limitations of well‐established techniques, we propose a novel work‐up of small skin specimens, combining so‐called three‐dimensional (3D) histopathology with bread loafing, while keeping orientation without artificial coloring. By inverting the tips of a narrow bread loafing, we obtain a compromise of both worlds. We regard the modality mostly suitable for evaluating small oval‐ or spindle‐shaped specimens containing tumors, especially where an otherwise full and elaborate margin control, such as the Tübinger Torte,^6^ would leave no or too little central tumor because of the small specimen size.

## 2 TECHNIQUE

At the Dermatology Department of the Medical University of Vienna at the Vienna General Hospital, we are often confronted with very small specimens from our surgery rooms because of excision from sensitive anatomic areas. Margin involvement is not rare in our cases, and an appropriate work‐up procedure therefore clinically relevant for single‐step excisions and delayed closure in both, in‐ and outpatient setting.

**FIGURE 1:**
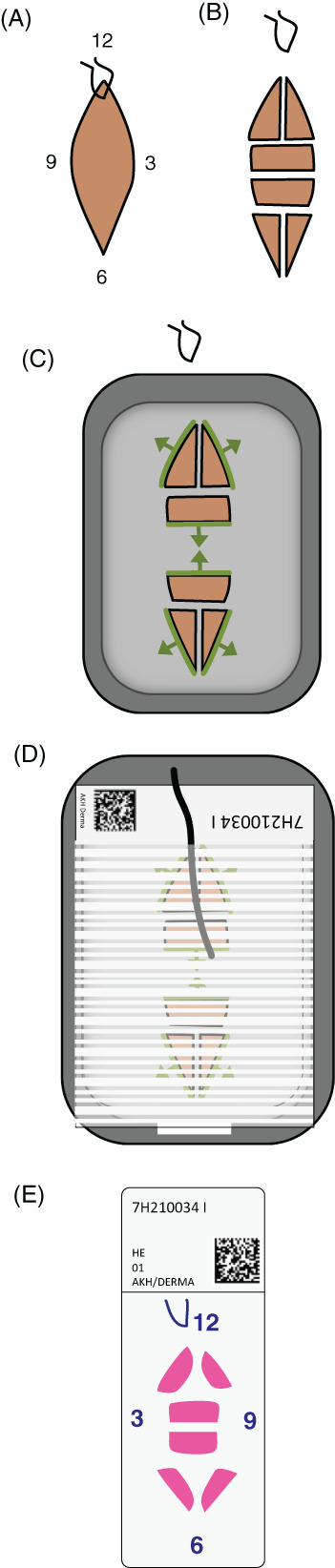
Conceptual steps for mixed 3D and bread loading work‐up without coloring. (A,B) The specimen is cut in one or two bread loafing sections in the center, the tips are split vertically. (C) The central cuts are oriented to the center, the tips oriented toward the outside to represent the outer resection margin. (D) The thread is melted into the back of the paraffin block to denote the original orientation. (E) The final slide allows to orient the full specimen without color reference

**FIGURE 2:**
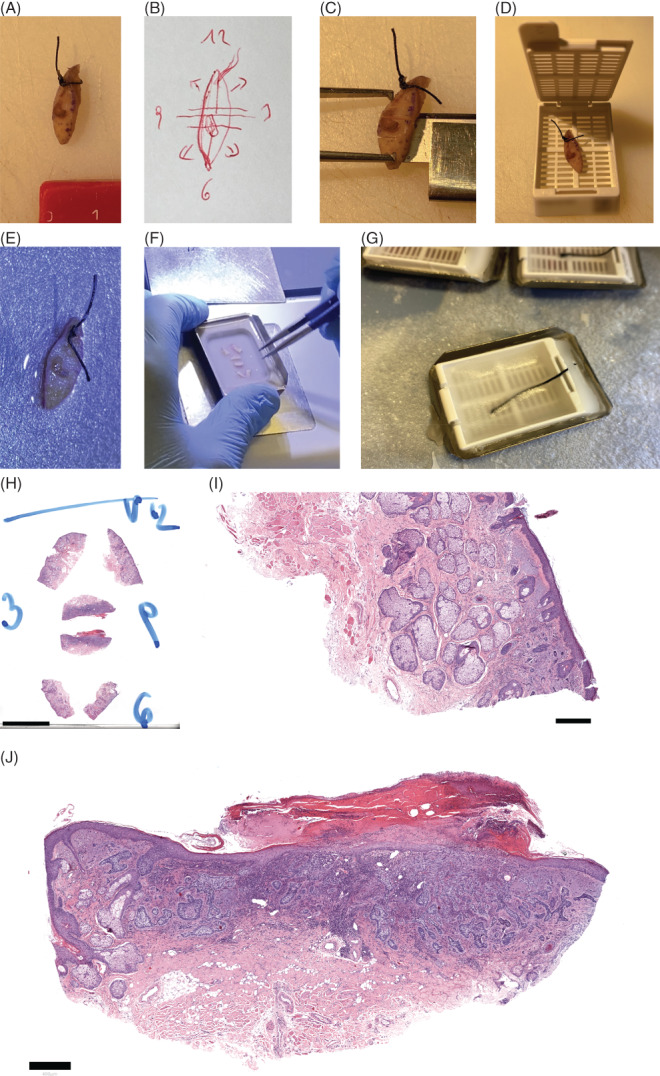
Tissue preparation for histopathological evaluation. (A) Small excision from the face with BCC as a clinical differential diagnosis. (B) Documented proposed work‐up sketch. (C) Pre‐incisions marking future sections before dehydration, keeping the full specimen—and thus orientation—intact. (D) The full specimen after fixation is forwarded to dehydration. (E) The dehydrated specimen on the next day, before embedding. (F) The split specimen with tips splitted and the outer resection margin on the bottom, and the inner sections oriented toward the center. (G) A thread is permanently inserted on the opposite side of the block, showing the original suture marking. (H) The final slide orientation of the specimen, showcasing a basal cell carcinoma with infiltrative growth, and involved lateral margins. The full orientation is intuitively visible for the complete specimen on one slide. Note that 3 and 9 o'clock are switched, as the slide depicts a horizontally flipped version of what is entered into the paraffin block (4x magnification). (I) Lateral resection margins can be reviewed as positive and allocated exact positions (40x magnification). (J) The central portion allows evaluation of the complete architecture, lateral margins at 3 and 9 o'clock, and the central basal resection margin (20x magnification)

During macroscopic work‐up by the pathologist (Figure 2A,B), the specimen is incised to ensure optimal dehydration conditions, while maintaining orientation through the process (Figure 2C,D). As part of the embedding (Figure 2D), the excision is divided using a scalpel and the individual cutting planes are aligned accordingly. The suture marking is removed, and the spindle tips are divided centrally (Figure 1A,B). The narrow cutting planes in the center of the specimen are tilted toward the lesion—representing the bread loaf of the technique—while the edges of the spindle tips are folded outward—enabling 3D assessment of the outer margins of the specimen (Figures 1C and 2F). Merging these techniques ensures examination of central tumor parts of small excisions, while providing increased coverage of lateral margins than bread loafing alone (Video S1).

To estimate the coverage of lateral margins, we calculated with a 2‐cm‐long spindle having at least 4‐mm lateral resection border, when conservatively assuming a non‐realistic straight form of 0‐cm width and depth. Given one central section with 4‐mm thickness and 4‐μm section width, approximately 80% of imaged lateral borders are retained, approximately 60% with two central sections

$$\left(4\;\mathrm{cm}-2\times \left[0.4\;\mathrm{cm}-0.0004\;\mathrm{cm}\right]\right)÷4\;\mathrm{cm}\approx 80\%.$$

This can be improved by making the central section as narrow as possible. Comparing, only doing 4‐mm bread loafing sections would image approximately 0.1% of the lateral margins

$$\left([2\;\mathrm{cm}÷0.4\;\mathrm{cm},],\times ,2,\times ,0.0004,\;,\mathrm{cm}\right)÷4\;\mathrm{cm}\approx 0.1\%.$$

Furthermore, we propose a technique to keep the orientation of the specimen throughout processing without coloring tissue. We achieve this by keeping the specimen intact and therefore oriented during initial grossing. By leaving the surgical suture marking in place and not dismantling the excision during macroscopy, we can avoid artificial coloring of the tissue (Figure 2E). After dehydration, the thread is removed as part of the embedding process, where it is afterward placed onto the top of the final paraffin block at the site of the original suture marking to enable future orientation (Figures 1D,E and 2G). With this approach, we can ensure instant excellent evaluability by dermatopathologists, without the need to memorize any sketches or coloring schemes. Because of the remaining risk of artifactual discontinuations of specimen, one needs to implement consistent quality control during workup and have awareness of reporting physicians, or still opt for coloring to identify true surgical margins.

## 3 DISCUSSION

To the best of our knowledge, there is no strong evidence of the superiority of a certain histopathologic work‐up technique in the assessment of NMSC in sensitive anatomic areas because all established techniques have limitations. Because of the often very small tissue size, 3D techniques may not allow comprehensive assessment of architecture and growth pattern of the tumor within the center. In addition, sectioning of the margins needs to be done with due care when sampling lateral borders starting from the outside. If too many sections are cut off before retrieving tissue for H&E staining, truly tumor‐free margins may be misinterpreted as tumor‐infiltrated margins. In contrast, bread loafing allows visualization of the distance between the tumor and the surgery margins in visualized planes and enables a more comprehensive assessment of the central lesion. On the other hand, coverage here is highly dependent on the width of the tissue incisions, and the number of sequential sections made when cutting the formalin‐fixed paraffin‐embedded block. So, while bread loafing enables optimal evaluation of tumor architecture and pattern, there is low explicit coverage of lateral margins, especially the tips. Three‐dimensional work‐up guarantees histopathological margin control, which can be particularly difficult in narrow specimens, as this often leaves the central parts containing the tumor disarrayed, thus hindering evaluation of architecture and pattern. We, therefore, propose to combine these approaches to reduce deficits and use the strengths of the individual methods. This proposed technique works under the assumption that basal incomplete resections happen most commonly within the center, which may not hold true for all cases. As a downside, it also demands consistent dexterity of the technician, as well as standardized sketching and embedding within a laboratory. Nevertheless, by merging techniques, we create in our view an optimized version of both, thereby holding the potential to minimize the risk of recurrence and avoid unnecessary removal of healthy tissue.

The proposal of combination is based on logical estimations. It remains to be shown from follow‐up studies comparing work‐up techniques, whether for example, the increased coverage of tips compared to conventional bread loafing translates to improved clinical outcomes.

## CONFLICT OF INTEREST

The authors declare that there are no conflict of interests.

## Supporting information


**Video S1**: Supporting Information
